# Prediction Model of Tumor Regression Grade for Advanced Gastric Cancer After Preoperative Chemotherapy

**DOI:** 10.3389/fonc.2021.607640

**Published:** 2021-04-15

**Authors:** Wei Xu, Qianchen Ma, Lingquan Wang, Changyu He, Sheng Lu, Zhentian Ni, Zichen Hua, Zhenglun Zhu, Zhongyin Yang, Yanan Zheng, Runhua Feng, Chao Yan, Chen Li, Xuexin Yao, Mingmin Chen, Wentao Liu, Min Yan, Zhenggang Zhu

**Affiliations:** ^1^ Department of General Surgery, Shanghai Key Laboratory of Gastric Neoplasms, Shanghai Institute of Digestive Surgery, Ruijin Hospital, Shanghai Jiao Tong University School of Medicine, Shanghai, China; ^2^ Department of Pathology, Ruijin Hospital, Shanghai Jiao Tong University School of Medicine, Shanghai, China

**Keywords:** advanced gastric cancer, preoperative chemotherapy, tumor regression grade, prediction model, survival

## Abstract

**Background:**

Preoperative chemotherapy (PCT) has been considered an important treatment for advanced gastric cancer (AGC). The tumor regression grade (TRG) system is an effective tool for the assessment of patient responses to PCT. Pathological complete response (TRG = 0) of the primary tumor is an excellent predictor of better prognosis. However, which patients could achieve pathological complete response (TRG = 0) after chemotherapy is still unknown. The study aimed to find predictors of TRG = 0 in AGC.

**Methods:**

A total of 304 patients with advanced gastric cancer from July 2009 to November 2018 were enrolled retrospectively. All patients were randomly assigned (2:1) to training and internal validation groups. In addition, 124 AGC patients receiving PCT from December 2018 to June 2020 were included prospectively in the external validation cohort. A prediction model for TRG = 0 was established based on four predictors in the training group and was validated in the internal and external validation groups.

**Results:**

Through univariate and multivariate analyses, we found that CA199, CA724, tumor differentiation and short axis of the largest regional lymph node (LNmax) were independent predictors of TRG = 0. Based on the four predictors, we established a prediction model for TRG = 0. The AUC values of the prediction model in the training, internal and external validation groups were 0.84, 0.73 and 0.82, respectively.

**Conclusions:**

We found that CA199, CA724, tumor differentiation and LNmax were associated with pathological response in advanced gastric cancer. The prediction model could provide guidance for clinical work.

## Introduction

Gastric cancer (GC) causes enormous health and economic burdens worldwide. GC is the fifth most common cancer and the third leading cause of cancer-related deaths worldwide (1, 2). In China, GC is the second most common cancer and the second leading cause of cancer death (3). Despite the declining incidence of GC, patients still have poor prognosis. Gastric cancer is often either asymptomatic or may cause only nonspecific symptoms in its early stage. When patients experience symptoms, the cancer has often already reached an advanced stage with regional lymph node metastasis or distant metastasis. Surgery and chemotherapy are the main methods for the treatment of advanced gastric cancer (AGC). However, the prognosis of patients with AGC is still poor (4–6). Currently, preoperative chemotherapy (PCT) is considered a standard therapy for AGC (7–10). PCT has potential benefits, such as downstaging the tumor to increase the chance of curative resection while eliminating potential micrometastases to prevent or reduce tumor recurrence and metastasis and improving tumor-associated symptoms (11, 12). However, not all patients benefit from PCT. Patients respond to PCT differently. During preoperative chemotherapy, some patients do not respond, some have adverse events, and some progress and even lose the opportunity for radical surgery. The tumor regression grade (TRG) system is an effective histopathological evaluation method for assessing patient response to PCT. Based on the TRG system, some studies found that patients with a major pathologic response will have better overall survival than those with no response or minor pathologic changes after PCT in AGC (13–15). There are several TRG systems for the assessment of the tumor pathological response to PCT, including the Mandard, Ninomiya, Becker and Ryan classification systems (16–19). Different people have different TRG grades, and patients with pathological complete response postchemotherapy have a longer survival and better prognosis.

However, in East Asia, an area with a high incidence of gastric cancer, PCT has not become a routine treatment for AGC. Therefore, it is vital to select patients who would most likely benefit from PCT and find the most suitable patients to receive PCT. Thus, the purpose of this study is to explore predictors for pathological complete response and establish a prediction model for pathological complete response in AGC. Based on the prediction model, patients who would most likely benefit from PCT can be identified, and physicians can be more confident in recommending PCT to these patients. For this purpose, we conducted this retrospective-prospective study to explore potential predictors for pathological complete response in AGC and developed a prediction model to guide clinical application.

## Materials and Methods

### Patient Selection and Study Design

This study was divided into two parts. In the first part, we used retrospective data to construct the prediction model and carried out internal validation. In the second part, after the prediction model was established, we collected data prospectively and then conducted an external validation of the prediction model.

Gastric cancer patient data from Shanghai Ruijin Hospital were retrospectively collected from July 2009 to November 2018. All gastric cancer patients were confirmed by endoscopic biopsy. The inclusion criteria were as follows: 1. patients were pathologically confirmed as having gastric adenocarcinoma; 2. patients had successfully undergone PCT before surgery; 3. gastrectomy was performed after PCT; 4. TRG can be assessed; and 5. pretreatment clinicopathological data can be collected. Samples were excluded if the patient did not meet the inclusion criteria. Patients in the retrospective cohort were randomly assigned (2:1) to a training group and an internal validation group. We developed a prediction model for TRG = 0 in the training group and then verified it in the internal validation group.

After the prediction model was established, we prospectively collected and recorded data from AGC patients from December 2018 to June 2020. The inclusion and exclusion criteria were the same as the criteria in the retrospective cohort mentioned above. The prediction model was verified in this prospective external validation group.

### Assessment System for Tumor Regression Grade

This study applied the Ryan classification system, which is the most widely used and applied by the College of American Pathologists (CAP) and the Chinese Society of Clinical Oncology (CSCO), to assess the pathological response of tumors to PCT (10, 20). In the Ryan classification system, TRG is a semiquantitative parameter describing a relative proportion of residual tumor and stromal fibrosis. TRG of the primary tumor is divided into four categories: grade 0 (complete response: no viable cancer cells), grade 1 (moderate response: single cells or small groups of cancer cells), grade 2 (minimal response: residual cancer outgrown by fibrosis) and grade 3 (poor response: minimal or no tumor cells killed; extensive residual cancer). All histological slides were reexamined by the same pathologist to confirm the TRG grade.

### Data Collection and Statistical Analysis

Pretreatment clinicopathological factors with potential prediction value were collected, including sex, age, body mass index (BMI), hemoglobin, leukocyte, neutrophil, lymphocyte, thrombocyte, prealbumin, total protein, albumin, CA125, CA199, CA724, CEA, AFP, tumor location, tumor differentiation, signet ring cell carcinoma component, Bormann type, chemotherapy regimen and short axis diameter of the largest regional lymph node (LNmax). Tumor location was classified into proximal, middle, distal 1/3 and whole stomach. LNmax was measured using multi-detector-row computed tomography (MDCT). The survival time after gastrectomy for every patient was also recorded by follow-up. The last follow-up time was 30, November 2019.

Univariate analysis was used to investigate whether any clinicopathological factors were correlated with TRG = 0. A nonparametric Mann-Whitney rank test or t test was used for the analysis of quantitative data. The chi-square test was used to compare categorical data. For the potential predictors, which were originally continuous variables, we performed receiver operating characteristic (ROC) curve analysis using the observed outcomes (TRG = 0 vs TRG≠0) and identified an optimal cut-off value that maximized the area under the curve (AUC) of the ROC curve. Through multivariate stepwise logistic regression analysis, we further investigated independent predictors for TRG = 0. Based on the odds ratio (OR) of independent predictors, a prediction model for TRG = 0 was established. Thereafter, the prediction model was verified in the internal and external validation groups.

All statistical tests were two-tailed, and the differences were considered statistically significant at p values <0.05. Data analysis was conducted using SPSS software version 25 (IBM Statistical Product and Service Solutions, Armonk, USA). The ROC and K-M survival curves were constructed by GraphPad Prism Version 5 (GraphPad Software, USA).

## Results

### Survival Analysis of Different TRG Groups

Previous studies showed that complete response (TRG = 0) after PCT was a predictor of good prognosis (13–15). To confirm the prognostic value of TRG, we conducted survival analysis of different TRG groups in the retrospective cohort. The last follow-up date for patients in the retrospective cohort was 30, November 2019, and the median follow-up time was 36.73 months (range 0.50 - 110 months). Of the 304 patients in the retrospective cohort, 90 patients (29.61%) had died of GC by the last follow-up day. A total of 26 (8.55%) patients were lost during the follow-up period.

**Figure 1:**
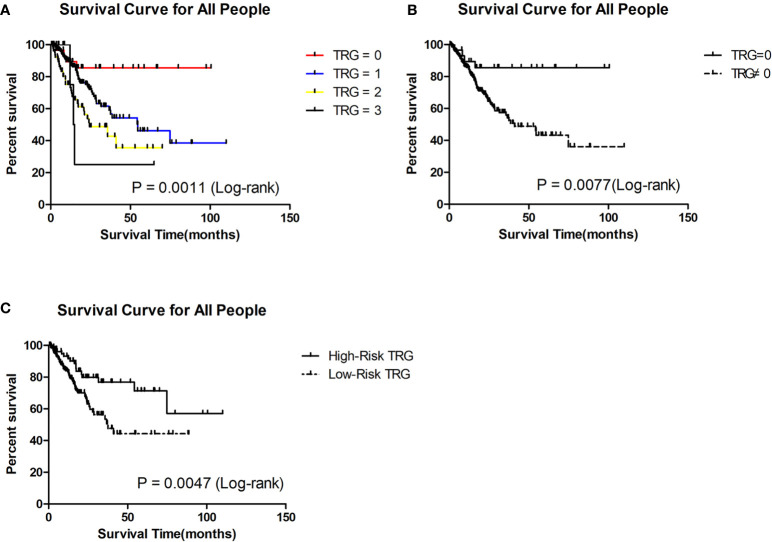
Survival analysis of patients in different TRG group in the retrospective cohort. **(A)** Survival analysis of patients between 0, 1, 2 and 3 grade TRG group. **(B)** Survival analysis of patients between TRG = 0 and TRG ≠0 group. **(C)** Survival analysis of patients between low-risk and high-risk TRG group.

Through the Kaplan-Meier (K-M) survival curve, we found that patients with TRG = 0 had significantly better survival than the others (P = 0.0011, **Figure 1A**). The estimated median survival of patients with TRG = 0, 1, 2, and 3 were undefined, 54.60, 24.40 and 14.64 months, respectively. The estimated 3-year survival rate of patients with TRG = 0 was significantly higher than that of patients with TRG≠0 (85.51% vs 54.13%, P=0.0077, **Figure 1B**).

**Figure 2:**
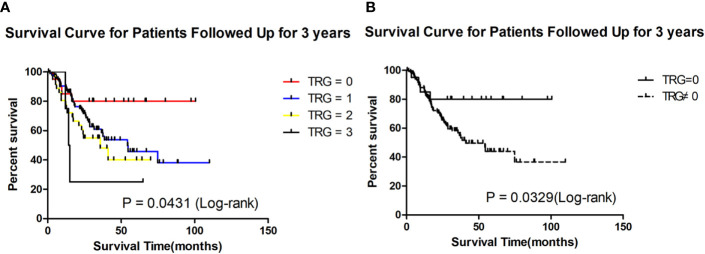
Survival analysis of patients followed up for 3 years in different TRG group in the retrospective cohort. **(A)** Survival analysis of patients between 0, 1, 2 and 3 grade TRG group. **(B)** Survival analysis of patients between TRG = 0 and TRG ≠0 group.

For 156 patients who were followed up for more than 3 years in the retrospective cohort, we also the plotted the K-M survival curve. The patients with TRG = 0 also had better survival than the others (P = 0.0431, **Figure 2A**). The median survival of patients with TRG = 0, 1, 2, and 3 were undefined, 54.30, 35.70 and 14.65 months, respectively. The 3-year survival rate of patients with TRG = 0 was also significantly higher than that of patients with TRG≠0 (80% vs 56.70%, P=0.0329, **Figure 2B**). Therefore, TRG = 0 was the focus of our attention, and we developed a prediction model for it.

### Characteristics of the Study Population

A total of 304 gastric cancer patients were enrolled retrospectively, and 124 patients were enrolled prospectively. All patients’ clinical stages at diagnosis were cT4N+Mx, which indicated that these tumors have invaded the serosal layer of the stomach and have regional lymph node metastasis, with or without distant metastasis. In the retrospective cohort, the study population comprised 224 male and 80 female patients. The median age was 61 years (range: 21-80 years). There were 281 patients without distant metastasis, 4 patients with single liver metastasis and 19 patients with retroperitoneal lymph node metastasis. All patients had received an average of three cycles of PCT before gastrectomy. The main regimens of PCT were EOX (Epirubicin plus Oxaliplatin and Capecitabine) and taxane-containing chemotherapy. There were 30 patients assessed with TRG = 0, including 6 patients with positive lymph nodes, which means that the primary tumor completely disappeared, but positive lymph nodes remained. Clinicopathological factors were compared between the different TRG groups (**Table 1**). We found that CA199, CA724, tumor differentiation, pathological type of signet ring cell carcinoma and LNmax were significantly different (P<0.05) between the different TRG groups. To develop a prediction model for TRG = 0, the study population was randomly assigned into the training set (202 patients) and the internal validation set (102 patients).

**Table 1:** Univariate analysis: Characteristics of the Whole Study Population.

Characteristics	Total (N = 304)	TRG = 0 (n = 30)	TRG ≠ 0 (n = 274)	P
**Sex (n[%])**				0.41 *
Male	224 (73.69)	24 (80.00)	200 (72.99)	
female	80 (26.31)	6 (20.00)	74 (27.01)	
**Age (y)**				0.51§
Median (range)	61(21-80)	61(31-75)	61(21-80)	
**BMI (kg/m^2^)**				0.58§
Median (range)	22.65(14-36.33)	22.72(19.53-31.74)	22.54(14-36.33)	
**Hemoglobin(g/L)**				0.45§
Median (range)	121(44-166)	115.50(52-162)	121(44-166)	
**Leukocyte(10^9/L)**				0.42§
Median(range)	5.80(2.40-19.70)	6.30(3.10-11.20)	5.80(2.40-19.73)	
**Neutrophil(10^9/L)**				0.53§
Median (range)	3.57(1.14-17.22)	3.97(1.59-8.27)	3.55(1.14-17.22)	
**Lymphocyte(10^9/L)**				0.28§
Median (range)	1.47(0.59-7.51)	1.51(0.63-2.77)	1.44(0.59-7.51)	
**Thrombocyte** **(10^9/L)**				0.30§
Median (range)	233(82-924)	248(93-924)	231(82-875)	
**Prealbumin (g/L)**				0.92*
Median (range)	203(79-354)	207(145-280)	203(79-354)	
**Total Protein(g/L)**				0.57§
Median (range)	64(46-80)	63.5(51-72)	64(46-80)	
**Albumin (g/L)**				0.46§
Median (range)	36(20-46)	37.50(27-44)	36(20-46)	
**CA125(U/mL)**				0.80§
Median (range)	12.85(3.80-361.30)	11.70(3.90-160.7)	13(3.80-361.30)	
**CA199(U/mL)**				0.02§
Median (range)	9.7(0.80-7424.00)	6.5(1.40-109.70)	10.25(0.80-7424.00)	
**CA724(U/mL)**				0.002§
Median (range)	3.67(0.06-300.00)	1.68(0.06-61.23)	4.18(0.20-300.00)	
**CEA(ng/mL)**				0.66§
Median (range)	2.73(0.50-4996.88)	2.92(0.79-82.15)	2.71(0.50-4996.88)	
**AFP(ng/mL)**				0.92§
Median (range)	2.69(0.65-16965.09)	2.63(1.08-3103.14)	2.70(0.65-16965.09)	
**Location (n[%])**				0.73*
Cardia	87(28.62)	10(33.33)	77(28.10)	
Body	96(31.58)	10(33.33)	86(31.39)	
Antrum	113(37.17)	10(33.33)	103(37.59)	
Whole stomach	8(2.63)	0(0)	8(2.92)	
**Differentiation (n[%])**				0.000*
Well	87(28.62)	19(63.33)	68(24.82)	
poor	217(71.38)	11(36.67)	206(75.18)	
**Signet ring cell (n[%])**				0.02*
Yes	57(18.75)	1(3.33)	56(20.44)	
No	247(81.25)	29(96.67)	218(79.56)	
**Borrmann (n[%])**				0.17*
I	9(2.96)	2(6.67)	7(2.55)	
II	12(3.95)	3(10.00)	9(3.28)	
III	257(84.54)	23(76.67)	234(85.40)	
IV	26(8.55)	2(6.67)	24(8.76)	
**Regimens (n[%])**				
EOX	237(77.96)	25(83.33)	212(77.37)	0.46*
Non EOX	67(22.04)	5(16.67)	62(22.63)	
Taxane	34(11.18)	2(6.67)	32(11.68)	0.41*
Non Taxane	270(88.82)	28(93.33)	242(88.32)	
**LNmax (cm)**				0.002§
Median (range)	1.27(0.49-5.05)	1.84(0.49-5.00)	1.18(0.50-5.05)	

*χ2 test (compares the counts of categorical responses between 2 or more independent groups).

^§^Mann-Whitney rank test (a nonparametric alternative to the 2 sample t test compares the means of 2 independent groups).

In the prospective cohort, there were 85 male and 39 female patients. The median age was 63 years (range: 27-79 years). There were 113 patients without distant metastasis, 4 patients with single liver metastasis, 5 patients with retroperitoneal lymph node metastasis, 1 patient with single pulmonary metastasis and 1 patient with ovarian metastasis. The main regimens of PCT were SOX (S-1 plus Oxaliplatin) and FLOT (Fluorouracil plus Leucovorin, Oxaliplatin, and Docetaxel). Nine patients were assessed with TRG = 0, including 1 patient with positive lymph nodes.

### Derivation of a Prediction Model for TRG = 0

In the training group, the five factors (CA199, CA724, tumor differentiation, pathological type of signet ring cell carcinoma and LNmax) were also significantly different (P<0.05) between the different TRG groups (**Table 2**). To perform multivariate logistic regression analysis with the five factors, we performed ROC analysis for originally continuous variables, including CA199, CA724 and LNmax. The optimal cut-off values for CA199, CA724 and LNmax were 10.90 U/ml, 3.19 U/ml and 1.535 cm, respectively. The sensitivity, specificity and AUC values of CA199 were 51.74%, 85% and 0.67 ± 0.05, respectively (95% CI: 0.57–0.77; P = 0.002). For CA724, the sensitivity, specificity and AUC values were 56.14%, 80% and 0.65 ± 0.05, respectively (95% CI: 0.55–0.76; P = 0.002). For LNmax, the sensitivity, specificity and AUC values were 62.28%, 78.95% and 0.66 ± 0.07, respectively (95% CI: 0.52–0.81; P = 0.001).

**Table 2:** Univariate analysis: Characteristics in the Training Set.

Characteristics	Total (N = 202)	TRG = 0 (n = 20)	TRG ≠ 0 (n =182)	P
**Sex (n[%])**				0.77 *
Male	147 (72.77)	14 (70.00)	133 (73.08)	
female	55 (27.23)	6 (30.00)	49 (26.92)	
**Age (y)**				0.81§
Median (range)	61(21-80)	61(40-75)	61(21-80)	
**BMI (kg/m^2^)**				0.24§
Median (range)	22.23(15.70-31.74)	22.77(19.53-31.74)	22.09(15.70-31.30)	
**Hemoglobin(g/L)**				0.45§
Median (range)	119(44-166)	113.50(65-154)	120.50(44-166)	
**Leukocyte(10^9/L)**				0.63§
Median(range)	5.83(2.40-16.48)	6.20(3.10-11.20)	5.80(2.40-16.48)	
**Neutrophil(10^9/L)**				0.68§
Median (range)	3.60(1.14-8.31)	3.96(1.59-8.27)	3.57(1.14-8.31)	
**Lymphocyte(10^9/L)**				0.54§
Median (range)	1.43(0.63-2.77)	1.47(0.63-2.77)	1.43(0.69-2.65)	
**Thrombocyte** **(10^9/L)**				0.29§
Median (range)	233(86-875)	247(93-531)	231(86-875)	
**Prealbumin (g/L)**				0.92*
Median (range)	197.5(79-354)	207(151-249)	194.5(79-354)	
**Total Protein(g/L)**				0.57§
Median (range)	64(48-79)	63(51-72)	64(48-79)	
**Albumin (g/L)**				0.65§
Median (range)	36(20-46)	37(27-43)	36(20-46)	
**CA125(U/mL)**				0.64§
Median (range)	13.30(3.80-185.70)	13.85(3.90-160.7)	13.20(3.80-185.70)	
**CA199(U/mL)**				0.01§
Median (range)	10.15(0.80-7424.00)	5.80(1.40-109.70)	12.05(0.80-7424.00)	
**CA724(U/mL)**				0.02§
Median (range)	3.41(0.06-239.40)	1.90(0.06-61.23)	3.78(0.20-239.40)	
**CEA(ng/mL)**				0.71§
Median (range)	2.84(0.50-4996.88)	2.90(0.79-72.67)	2.78(0.50-4996.88)	
**AFP(ng/mL)**				0.51§
Median (range)	2.69(0.90-10783.52)	2.22(1.08-3103.14)	2.79(0.90-10783.52)	
**Location (n[%])**				0.43*
Cardia	62(30.69)	9(45.00)	53(29.12)	
Body	64(31.68)	6(30.00)	58(31.87)	
Antrum	70(34.65)	5(25.00)	65(35.71)	
Whole stomach	6(2.97)	0(0)	6(3.30)	
**Differentiation (n[%])**				0.000*
Well	54(26.73)	13(65.00)	41(22.53)	
poor	148(73.27)	7(35.00)	141(77.47)	
**Signet ring cell (n[%])**				0.03*
Yes	35(17.33)	0(0)	35(19.23)	
No	167(82.67)	20(100)	147(80.77)	
**Borrmann (n[%])**				0.12*
I	6(2.97)	2(10.00)	4(2.20)	
II	3(1.49)	1(5.00)	2(1.10)	
III	174(86.14)	15(75.00)	159(87.36)	
IV	19(9.40)	2(10.00)	17(9.34)	
**Regimens (n[%])**				
EOX	158(78.22)	16(80.00)	142(78.02)	0.84*
Non EOX	44(21.78)	4(20.00)	40(21.98)	
Taxane	23(11.39)	2(10.00)	21(11.54)	0.84*
Non Taxane	179(88.61)	18(90.00)	161(88.46)	
**LNmax (cm)**				0.02§
Median (range)	1.34(0.49-5.05)	2.07(0.49-5.00)	1.20(0.50-5.05)	

*χ2 test (compares the counts of categorical responses between 2 or more independent groups).

^§^Mann-Whitney rank test (a nonparametric alternative to the 2 sample t test compares the means of 2 independent groups).

**Figure 3:**
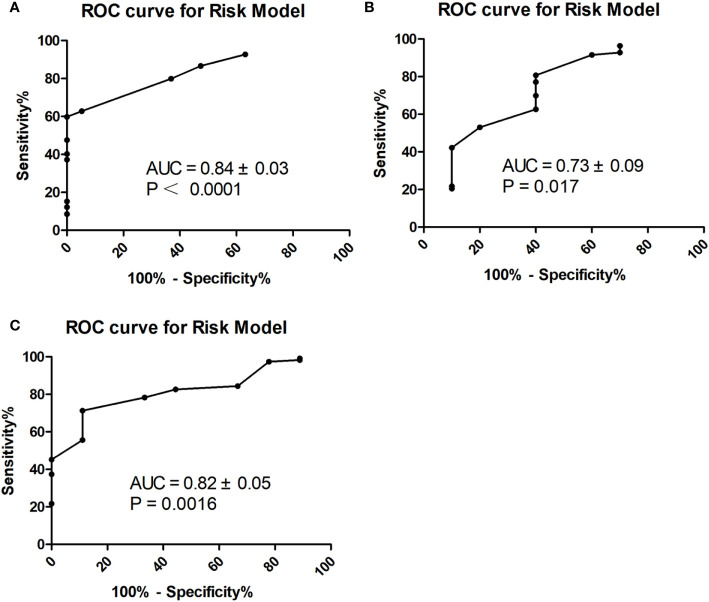
ROC curve for prediction model in the training **(A)**, internal **(B)** and external **(C)** validation group.

In multivariate logistic regression analysis, we found that four factors were significantly different (**Table 3**). CA199 ≤10.90 U/ml, CA724 ≤3.19 U/ml, well differentiation and LNmax ≥1.535 cm were independent predictors for TRG=0 (P<0.05). A risk score was assigned to each predictor on the basis of the OR resulting from the logistic regression analysis. CA199 ≤10.90 U/mL, CA724 ≤3.19 U/mL, well differentiation and LNmax ≥1.535 cm were assigned 5, 4, 7, and 7 points, respectively (**Table 4**). The final scores ranged from 0 to 23 points. Based on the risk score, we established a prediction model for TRG = 0. According to the prediction model, we calculated the sum scores of the patients in the training group. The AUC of the prediction model was 0.84 (SD = 0.03; 95% CI: 0.77-0.91; P<0.0001) (**Figure 3A**). The optimal cut-off point for TRG in the prediction model was 13 points resulting from ROC curve analysis. The patients were divided into a low-risk (≤13 points) and a high-risk (>13 points) TRG group. TRG = 0 was discovered in 0% and 22.35% of the patients in the low-risk and high-risk TRG groups, respectively. The higher the score is, the more likely it indicates TRG = 0.

**Table 3:** Multivariate analysis: variables correlated with TRG in the training set.

Variables	B	P**	OR	95% CI
**CA199, ≤/>10.90 U/mL**	1.725	0.023	5.615	1.272-24.782
**CA724, ≤/>3.19 U/mL**	1.511	0.029	4.531	1.167-17.589
**Differentiation, well/poor**	2.005	0.002	7.426	2.143-25.725
**Signet Ring cell Carcinoma, yes/no**	17.341	0.998	33985914	0.000-0.000
**LNmax, ≥/<1.535cm**	2.073	0.002	7.945	2.085-30.279

B, beta coefficient; OR, odds ratio; 95% CI, 95% confidence interval.

**multivariable logistic regression analysis.

**Table 4:** Risk Score of Prediction Model for TRG.

Predictors	Score			
	0	4	5	7
**CA199 (U/mL)**	>10.90		≤10.90	
**CA724 (U/mL)**	>3.19	≤3.19		
**Differentiation**	poor			well
**LNmax (cm)**	<1.535			≥1.535

### Validation of the Prediction Model for TRG = 0

To validate the prediction model for TRG = 0, we also performed ROC analysis in the internal and external validation groups. The AUC of the prediction model in the internal validation group was 0.73 (SD=0.09; 95% CI: 0.55-0.91; P=0.017) (**Figure 3B**). The sensitivity, specificity, negative predictive value (NPV), positive predictive value (PPV), and accuracy in the internal validation group were 60%, 77.11%, 94.12%, 24% and 75.27%, respectively. In the external validation group, the AUC of the prediction model was 0.82 (SD=0.05; 95% CI: 0.71-0.92; P=0.0016) (**Figure 3C**). The sensitivity, specificity, NPV, PPV, and accuracy in the external validation group were 55.56%, 82.61%, 95.96%, 20% and 80.65%, respectively.

### Prognostic Value of the Prediction Model

To evaluate whether the prediction model is useful for predicting prognosis, we plotted Kaplan-Meier (K-M) survival curves for the low-risk and high-risk TRG groups in the retrospective cohort. We found that patients in the high-risk TRG group had better survival than patients in the low-risk TRG group (P<0.05, **Figure 1C**). The estimated median survival times of patients in the high- and low-risk TRG groups were undefined and 37.50 months; the estimated 3-year survival rates were 76.75% and 53.45%, respectively. Therefore, this prediction model was also effective for prognosis.

## Discussion

Currently, PCT is considered a standard therapy for AGC. However, the risk of tumor progression is still worrisome. Despite enduring the side effects of PCT, patients may still experience tumor progression and lose the chance of radical surgery (21). The TRG system is an effective assessment method for pathological response and has been widely applied in clinical work. In the current study, we applied the Ryan classification system to assess tumor response after PCT. AGC patients have different responses to PCT. Complete response (TRG = 0) was considered the best response and a predictor of better prognosis. To explore which patients will benefit most from PCT, we conducted this current study. We found that four pretreatment factors were independent predictors for TRG=0 in AGC and established a prediction model for it. The four predictors were CA199 ≤10.90 U/mL, CA724 ≤3.19 U/mL, LNmax ≥1.535 cm, and well differentiation of the tumor. The four indicators used in this model are easy to obtain, which extends the model’s range of applications.

Tumor markers (CA125, CA199, CA724, CEA and AFP) are widely used for the early diagnosis and prognostic evaluation of gastric cancer (22–24). However, the clinical value of tumor markers for predicting the response to PCT is still unclear (25, 26). A previous study showed that high preoperative CA724 and CA199 levels were associated with a higher risk of death, and a decrease (>70%) in CA724 may predict the pathological response to PCT (26). In our study, we found that CA199 ≤10.90 U/mL and CA724 ≤3.19 U/mL were associated with TRG = 0 in GC patients after PCT. In other words, lower CA199 and CA724 levels are associated with a better response to PCT.

Patients with well-differentiated GC are regarded to have better survival than those with poorly differentiated GC (27, 28). In addition, a previous study showed that well differentiation is a vital predictor of pathologic response (29). In this study, we also found that well differentiation is an independent predictor of TRG = 0. This is consistent with previous studies.

In the current study, LNmax was significantly associated with TRG = 0. To our knowledge, this is the first report showing the value of LNmax for predicting TRG = 0 after PCT in gastric cancer. We speculate that patients with large regional lymph nodes have strong immunity against infection and tumor cell invasion. Patients with large regional lymph nodes have the chance to respond sooner to the tumor, with a stronger response. With stronger immunity, the effect of PCT is increasingly obvious. These patients will have a better pathological response and prognosis. However, further investigations are needed to identify the underlying physiological mechanism.

We established a prediction model for TRG = 0 after PCT in advanced gastric cancer with four independent predictors. The prediction model showed that patients with higher scores were more likely to obtain a better pathological response and prognosis. We found that the optimal cut-off value of the prediction model was 13 points. If patients received more than 13 points, we recommend that these patients receive PCT instead of direct surgery. On the other hand, if patients received very low points, we should be careful in choosing PCT for these patients.

This study had some limitations. This was a single-center clinical study, and the sample size was not very large. Patients in this study were enrolled over a large time span (2009-2018) and had different chemotherapy regimens. Besides, the model was based on common clinicopathological factors. Currently, the Cancer Genome Atlas (TCGA) Research Network conducted a comprehensive molecular characterization of GC, which proposed a molecular classification dividing GC into four subtypes: tumors positive for Epstein-Barr virus (EBV), microsatellite instability (MSI) tumors, genomically stable (GS) tumors and tumors with chromosomal instability (CIN) (30). Many studies had been carried out on the basis of TCGA classification. Several studies showed that patients with MSI-low GC could benefit from chemotherapy plus surgery, however those with MSI-high GC did not (31–33). Besides, several studies showed EBV positivity in GC patients was associated with better prognosis (34, 35).To improve this prediction model, we could integrate genetic factors according to the TCGA classification in future studies. Further investigation is needed to identify other predictors and optimize the prediction model for TRG = 0.

Despite the limitation of this study, we found that CA199, CA724, tumor differentiation and LNmax were associated with pathological response in AGC patients. We established a prediction model for TRG = 0 in AGC patients that could provide guidance for clinical work.

## Data Availability Statement

The raw data supporting the conclusions of this article will be made available by the authors, without undue reservation.

## Ethics Statement

The studies involving human participants were reviewed and approved by the Human Ethics Committee of Shanghai Jiao Tong University School of Medicine Ruijin Hospital. The patients/participants provided their written informed consent to participate in this study.

## Author Contributions

WX conceived and designed the study, analyzed the data and wrote the paper. QM reexamined the tumor regression grade of all patients. LW, CH, SL, ZN, ZH, ZLZ, ZY and YZ collected the pretreatment clinicopathological factors of all patients. RF, CY, CL, XY and MC followed up the patient’s survival status. WL revised the paper. MY and ZGZ gave professional guidance. All authors contributed to the article and approved the submitted version.

## Funding

This work was supported by National Natural Science Foundation of China NO. 81772518 and clinical research project of Ruijin Hospital, Shanghai Jiao Tong University School of Medicine (DLY201602 and 2018CR003).

## Conflict of Interest

The authors declare that the research was conducted in the absence of any commercial or financial relationships that could be construed as a potential conflict of interest.
